# Construction Notes

**Published:** 1895-01-12

**Authors:** 


### CONSTRUCTION NOTES.

**New Hospital at Berkeley.**

The commodious house and premises recently purchased by Lord Fitzhardinge for the Berkeley Hospital, situate in Maryport Street, Berkeley, Gloucestershire, have been throughly repaired, renovated, and altered to suit the requirements of the hospital, by the generosity of his lordship. On the ground floor there is a spacious entrance hall, committee-room, matron's-room, day-room for patients, 19 ft. by 16 ft., large kitchen, and offices, and a mortuary has been erected at the back. On the first floor is a roomy landing, men's ward, 22 ft. by 16 ft., women's ward, and a special ward for accidents; a matron's bed-room, bathroom, with hot and cold water, for the use of the patients; the rooms are lofty, and fitted with an excellent arrangement of gas fittings. The second floor contains bed-rooms for the officials and servants. There is a pleasure garden and kitchen garden at the rear; the drains are fitted with patent traps, and the sanitary arrangements are complete in every way. The work has been satisfactorily carried out by Mr. F. W. Ford, of Uley, under the superintendence of Mr. R. A. H. Ransford, at a cost of about £300.

**Swanage New Cottage Hospital.**

This cottage hospital, which it is predicted will prove a great boon to the neighbourhood, was erected in memory of the late Mr. and Mrs. Burt, whose names are known as untiring local benefactors, the necessity of a memorial of whom has now been universally recognised. The proposed site of this memorial is in the Queen's Road, on the Durslton Park Estate. Accommodation will be provided for twelve patients. The building will be of dark and red brick. The whole of the wards are to be on the ground floor, with a large hall in the centre of the building, which will be lighted from the roof, and have an airy and comfortable appearance. The floors will be of wood blocks, and the heating will be by fireplaces and pipes. Every care is to be exercised with respect to sanitation, and the best sanitary appliances will be used. On entering the ground floor the accident ward will be immediately on the right, and leading from this, still further to the right, will be the men's ward. Behind the last will be a surgery, bath-room, &c. The room on the left of this will be set apart for the matron, while adjoining it will be the women's ward. At the rear of this a commodious day-room will be provided, from which access can be obtained to a verandah. Between the suites of rooms will be the great hall, behind which various domestic offices are established. The fireplace in the great hall will no doubt be of a special character, as it is proposed to place in connection with it an inscription setting forth the fact that the institution was erected to the memory of the late Mr. W. Burt. On the top floor of the building provision has been made for matron's and servants' bed-rooms, also a room which may be utilised for the benefit of private patients if required. Another room is also designed, which is to be placed at the disposal of the parochial nurses. The plans of the proposed hospital were drawn by Mr. Walter Fletcher, of Wimborne.

**Rotherham Hospital and Dispensary Improvements.**

Two great improvements have been effected in this hospital by the committee during the last quarter year. Experience having proved, in the opinion of the medical staff, that hot-water pipes were needed to be supplied to all the wards. The wards were so cold during the past winter that special provision of extra stores had to be made for the sake of the patients. A contract has accordingly been made with Messrs. Newton, Chambers, and Co. for laying down the additional pipes and the provision of the boiler. The total cost has amounted to £125. The other chief improvement has concerned a thorough examination and reconstruction of the drainage of the hospital. For this purpose the services of the borough surveyor, Mr. G. Jennings, had been engaged, and the drainage now of the institution has been rendered as perfect as possible.

